# Differences of physical activity, sleep quality and depressive symptoms in patients with diabetes and non-diabetes undergoing maintenance hemodialysis in Xi'an, China: A cross-sectional study

**DOI:** 10.1016/j.pmedr.2026.103641

**Published:** 2026-09-22

**Authors:** Lu Zhang, Sumei Zhang

**Affiliations:** School of Nursing and Rehabilitation, Xi'an Medical University, NO.1 Xinwang Road, Weiyang district, Xi'an, Shaanxi 710021, PR China

**Keywords:** Renal Dialysis, Diabetes mellitus, Exercise, Sleep quality, Depressive symptoms

## Abstract

**Objective:**

To compare the physical activity, sleep quality and depressive symptoms between patients with diabetes and non-diabetes undergoing maintenance hemodialysis, and identify associated influencing factors.

**Methods:**

A descriptive cross-sectional design was adopted. Maintenance hemodialysis patients were enrolled from two hemodialysis centers in Xi'an, China between April and May 2022. Sociodemographic and clinical data were collected, with physical activity assessed via Human Activity Profile, sleep quality via Pittsburgh Sleep Quality Index, and depressive symptoms via Beck Depression Inventory-II.

**Results:**

Of 286 participants, 113 had diabetes and 173 did not. Diabetic patients had lower adjusted activity scores (40.07 ± 22.94 vs. 55.94 ± 21.35, *P* < 0.01), higher PSQI global scores (9.04 ± 4.48 vs. 7.15 ± 4.47, *P* < 0.01), and higher BDI-II scores (17.32 ± 10.75 vs. 12.95 ± 9.26, *P* < 0.01) than non-diabetic patients. Multivariate logistic regression showed that age (OR = 1.02, 95% CI: 1.01, 1.04) and BDI-II score (OR = 1.04, 95% CI: 1.01, 1.07) were positively associated with diabetes, whereas AAS (OR = 0.98, 95% CI: 0.97, 0.99) and MHD duration (OR = 0.99, 95% CI: 0.98, 1.00) were negatively associated.

**Conclusions:**

Diabetic patients receiving maintenance hemodialysis exhibit poorer physical activity levels, sleep quality, and depressive symptoms compared with non-diabetic counterparts. Accordingly, routine combined screening of physical activity, sleep disturbance and depressive symptoms should be embedded into daily dialysis nursing.

## Highlights

• Diabetic hemodialysis patients have lower physical activity than non-diabetics.
• They also experience worse sleep quality and more depressive symptoms.
• Routine screening for these three conditions is recommended in dialysis care.

## 1 Introduction

Chronic kidney disease is a global public health issue that can lead to kidney failure and cardiovascular complications (Jankowski et al., 2021). As the main treatment method for renal failure, maintenance hemodialysis treatment not only brings changes to patients' lives, but also poses emotional and physical challenges to patients (Rota-Musoll et al., 2020). On the basis of improving patient survival rate, it is also an important research topic to adjust the physical and psychological status of patients and improve their quality of life.

Due to the influence of the primary disease of uremia, the restriction of physical activity by dialysis treatment and the sedentary lifestyle, the physical activity ability of patients can be reduced and muscle consumption can be increased (Jamshidpour et al., 2020). Many studies have shown that patients undergoing maintenance hemodialysis (MHD) can benefit from physical activity, and many indicators related to health and quality of life all can be improved (Morgan et al., 2022).

Sleep disorder is very common in patients on dialysis. Regardless of the primary disease of end stage renal disease in patients on dialysis, the incidence of sleep disorder is higher than that in the general population. It is reported that about 70% -80% of patients on dialysis have sleep disorder (Al-Ali et al., 2021). Sleep disorder is also a very common symptom of patients with diabetes, which can affect the blood glucose regulation and quality of life of patients (Zhu et al., 2018).

Depressive symptoms are the most common mental and psychological problem in patients with end stage kidney disease (ESRD). And the incidence rates and severity of depression are markedly higher in ESRD patients with diabetes mellitus (Jeffery et al., 2021).This may be because the ESRD patients with diabetes have greater burden of complications and complexity of treatment, which may increase the psychological burden of patients and hinder emotional adjustment (Yoong et al., 2017). However, there are few studies on the depressive symptoms of patients with diabetes undergoing maintenance hemodialysis. It's necessary to evaluate the psychological condition to develop efforts to improve patients' depressive symptoms and outcomes.

Patients with diabetes undergoing maintenance hemodialysis often have wide-ranging complications, so the management of these patients is complex due to systemic complications and unstable clinical conditions. Based on our knowledge, there are few studies specifically evaluating physical activity, sleep quality and depressive symptoms in the patients with diabetes undergoing maintenance hemodialysis. The primary objective of this study was to compare physical activity levels, sleep quality, and depressive symptoms between maintenance hemodialysis patients with and without diabetes. The secondary objective was to identify factors associated with diabetes status in this population. We hypothesized that diabetic hemodialysis patients would exhibit lower physical activity, poorer sleep quality, and more severe depressive symptoms compared with their non-diabetic counterparts.

## 2 Methods

### 2.1 Study design and population

A descriptive cross-sectional study using convenience sampling was conducted to examine the levels of physical activity, sleep quality, and depressive symptoms in patients receiving maintenance hemodialysis from two hemodialysis centers in Xi'an, China, between April and May 2022, with comparisons made between those with and without diabetes mellitus. Diabetes mellitus was defined based on documented physician diagnosis in the electronic medical records.

The sample size was determined based on the empirical rule of thumb for regression analyses, which recommends 10 to 15 cases per independent variable (Peduzzi et al., 1996). With 17 independent variables considered in the logistic regression model and using 10–15-fold criterion, the required sample was calculated as 170–255. To account for potential incomplete responses, this estimate was inflated by 20%. The sample size was calculated to be 204–306. The final analytical sample of 286 participants exceeded this requirement.

### 2.2 Measures

#### 2.2.1 Inclusion and exclusion criteria

Eligible participants met the following inclusion criteria: (1) receiving regular maintenance hemodialysis for a minimum of three months; (2) aged 18 years or older; (3) able to provide written informed consent for voluntary participation.

Individuals were excluded based on the following criteria: (1) those who declined participation or refused to sign the informed consent after full explanation of the study procedures; (2) participants under 18 years of age; (3) patients unable to understand questionnaire items and complete relevant assessments independently even with assistance.

#### 2.2.2 Instruments

##### 2.2.2.1 Sociodemographic and clinical characteristics

Participants' sociodemographic profiles encompassed age, gender, marital status, and primary renal diagnosis, along with other relevant baseline characteristics. Blood pressure measurements were obtained prior to each hemodialysis session. Laboratory indicators including hemoglobin, serum calcium, and serum phosphate were collected at the time of participants' enrollment in the research.

##### 2.2.2.2 Evaluation of sleep quality

Sleep quality was evaluated using the Pittsburgh Sleep Quality Index (PSQI). This validated instrument consists of seven dimensions: subjective sleep quality, sleep latency, sleep duration, habitual sleep efficiency, sleep disturbances, use of sleep medication, and daytime dysfunction. The scale comprises 19 items in total, with overall scores ranging from 0 to 21; higher total scores correspond to poorer sleep quality. Participants were instructed to recall their sleep patterns during the month prior to enrollment when completing all questionnaire items. Respondents were categorized into poor sleepers (PSQI >5) and good sleepers (PSQI ≤5) (Buysse et al., 1989). In the present sample, the Cronbach's alpha for the PSQI was 0.82.

##### 2.2.2.3 Evaluation of physical activity

The Human Activity Profile (HAP) serves as a validated questionnaire for evaluating functional activity levels and physical functioning in daily life. This scale consists of 94 items corresponding to daily activities, which are sequenced from low to high based on the energy expenditure required for each task (Johansen et al., 2001).Item content ranges from basic movements, such as independent sitting and rising from a bed or chair (item one), to high-intensity exercise, including running three miles within 30 min (item 94). When completing the scale, participants were required to indicate their current status for each activity: actively performed, previously discontinued, or never undertaken. The Maximal Activity Score (MAS) reflects the total number of activities an individual can still complete at their peak aerobic capacity. Additionally, the Adjusted Activity Score (AAS) is calculated by subtracting discontinued high-energy activities from the MAS. This refined scoring approach enables a more accurate assessment of participants' functional activity ranges and the severity of their mobility limitations (Johansen et al., 2001). In the present sample, the Cronbach's alpha for the HAP was 0.98.

##### 2.2.2.4 Evaluation of depressive symptoms

The Beck Depression Inventory-II (BDI-II) consists of 21 items, with higher overall scores reflecting more severe depressive symptoms. Scores at or below 13 indicate minimal depressive severity; values ranging from 14 to 19 correspond to mild Depressive Symptoms. Scores between 20 and 28 represent moderate depressive status, while total scores from 29 up to 63 are classified as severe depressive symptoms (Beck et al., 1996). It is important to note that BDI-II scores reflect the severity of depressive symptoms. In the present sample, the Cronbach's alpha for the BDI-II was 0.93.

#### 2.2.3 Data collection

Data were collected through face-to-face interviewer-administered questionnaires and electronic medical records. Trained research assistants read each question to participants and recorded their responses to ensure comprehension and completeness, particularly for participants with visual or literacy difficulties. Questionnaires were administered during the middle phase of the 4-hour hemodialysis session, after patients had stabilized, to avoid pre-dialysis anxiety and post-dialysis fatigue.

#### 2.2.4 Ethical consideration

This research involved human participants and adopted a non-interventional design; no clinical trials or experimental interventions were performed on enrolled patients. Only routine physiological and psychological evaluation indicators were collected for analytical purposes.

All procedures involving human participants complied with the ethical requirements of the affiliated institutional review board and followed the principles outlined in the 1964 Helsinki Declaration, together with all subsequent revisions and relevant international ethical guidelines. The present investigation received official ethical approval from the Medical Ethics Review Committee of Xi'an Medical University (Approval No. XYLS2024220).

Written informed consent was obtained from all participants before enrollment, and participation remained entirely voluntary throughout the research process. This study posed no physical discomfort or health risks to participants; the only requirement was completion of questionnaires during their routine dialysis sessions. No identifiable individual information will be included in any published article derived from this study.

### 2.3 Statistical analysis

All statistical outcomes were presented as mean ± standard deviation for continuous data or percentages for categorical variables, depending on data distribution. Independent sample *t*-tests or Mann–Whitney *U* tests were adopted to compare physical activity, sleep quality, and depressive symptoms between maintenance hemodialysis patients with and without diabetes. The chi-square test was applied for intergroup comparisons of categorical indicators. Binary logistic regression was performed to identify factors associated with the presence of diabetes among maintenance hemodialysis patients. The dependent variable was diabetes status (yes vs. no). Independent variables included all factors that showed significant between-group differences in univariate analyses. This approach was intended to complement the primary descriptive comparison, not to establish prediction or causation, with results reported as odds ratios (OR) alongside their corresponding 95% confidence intervals (95%CI). For the scale measures, only total scores were used as independent variables to avoid multicollinearity among subdomains: the PSQI global score, the BDI-II total score, and the AAS from the HAP (the MAS was excluded because of its high correlation with the AAS). Multicollinearity among the final set of independent variables was assessed using the variance inflation factor (VIF); all VIF values were below 2 (ranging from 1.051 to 1.631), indicating no substantial multicollinearity. A two-tailed statistical significance threshold was set at *P* < 0.05. All data analyses were conducted using SPSS™ 24.0 software (Chicago, IL, USA).

## 3 Results

### 3.1 Descriptive data

A total of 313 patients were initially assessed, of whom 27 were excluded due to substantial missing data, resulting in a final sample of 286 participants in this cross-sectional investigation in Xi'an, China.

The participants had a mean age of (57.49 ± 15.97) years, and male individuals accounted for 66.78% of the total sample. In terms of educational background, 65.03% of the patients attained a college or higher education level. Regarding employment status, 81.46% of the enrolled subjects were unemployed. Additionally, diabetic nephropathy was identified as the primary cause of end-stage renal disease (ESRD) in 39.51% of all participants.

### 3.2 Main results

#### 3.2.1 Comparison of general characteristics between patients with diabetes and non-diabetes undergoing maintenance hemodialysis

Compared with participants without diabetes, those with diabetic comorbidity were significantly older (*P* < 0.01), presented with higher systolic and diastolic blood pressure values (*P* < 0.01, respectively), and exhibited a shorter duration of dialysis treatment (*P* < 0.01). In addition, the diabetic group showed a lower proportion of individuals maintaining regular physical activity (*P* = 0.01), along with higher percentages of married participants and retired cases (both *P* = 0.03 and *P* < 0.01, correspondingly) (Table 1).

**Table 1:** Comparison of general characteristics between patients with diabetes and non-diabetes undergoing maintenance hemodialysis (Xi'an, China; from April–May 2022).

	Total (*n* = 286)	Patients with Diabetes (*n* = 113)	Patients with Non-diabetes (*n* = 173)	*t*/*Z*/*χ*^2^	*P*
Age, years	57.49 ± 15.97	62.86 ± 12. 78	53.88 ± 16.86	−4.47	<0.01
Gender, n (%)				0.01	0.91
male	191 (66.78)	75 (66.37)	116 (67.05)		
female	95 (33.22)	38 (33.63)	57 (32.95)		
Education, n (%)				0.41	0.52
High school and below	186 (65.03)	76 (67.26)	110 (63.58)		
College and above	100 (34.97)	37 (32.74)	63 (36.42)		
Marriage, n (%)				4.52	0.03
married	97 (33.92)	83 (73.45)	106 (61.27)		
other(single, divorce, widowed)	189 (66.08)	30 (26.55)	67 (38.73)		
Employment, n (%)				22.33	<0.01
working	53 (18.53)	15 (13.27)	38 (21.97)		
retired	102 (35.66)	59 (52.21)	43 (24.86)		
other(unemployed、students)	131 (45.80)	39 (34.51)	92 (53.18)		
Exercise status, n (%)				6.56	0.01
Exercise	173 (60.49)	58 (51.33)	115 (66.47)		
No exercise	113 (39.51)	55 (48.67)	58 (33.53)		
MHD duration, months	35.44 ± 30.01	29.03 ± 22.38	39.64 ± 33.50	2.96	<0.01
BMI	21.82 ± 3.38	22.04 ± 3.15	21.94 ± 3.37	−1.32	0.19
SBP(mmHg)	150.97 ± 18.33	154.78 ± 16.96	148.49 ± 18.80	−2.98	<0.01
DBP(mmHg)	88.33 ± 49.95	91.30 ± 77.87	86.39 ± 13.26	−4.07	<0.01
Ca (mmol/L)	1.99 ± 0.45	2.03 ± 0.36	1.96 ± 0.50	−0.90	0.37
P (mmol/L)	1.82 ± 0.58	1.76 ± 0.58	1.86 ± 0.58	−1.04	0.30
Hb (g/L)	100.28 ± 22.83	99.29 ± 23.63	100.91 ± 22.37	0.51	0.61

(MHD: maintenance hemodialysis; SBP: systolic blood pressure; DBP: diastolic blood pressure; Ca: Serum calcium; P: Serum phosphorus; Hb: Hemoglobin.)

(*P*-values were obtained from chi-square tests for categorical variables and *t*-tests or Mann‑Whitney *U* test for continuous variables.)

#### 3.2.2 Comparison of physical activity differences between patients with diabetes and non-diabetes undergoing maintenance hemodialysis

Data did not conform to normal distribution, so Mann–Whitney *U* tests were used to compare the differences of physical activity between patients with diabetes and non-diabetes undergoing maintenance hemodialysis. In physical activity differences, there were significant differences in the scores of each dimension and total score between two groups. Compared with the patients with non-diabetes, patients with diabetes have lower scores of physical activity (*P* < 0.01) (Table 2).

**Table 2:** Comparison of physical activity differences between patients with diabetes and non-diabetes undergoing maintenance hemodialysis (Xi'an, China; from April–May 2022).

Characteristic	Patients with diabetes (n = 113)	Patients with Non-diabetes (n = 173)	*Z*	*P*
**MAS(**$\bar{x}$**±s)**	56.06 ± 21.30	67.99 ± 17.82	−5.21	<0.01
**AAS(**$\bar{x}$**±s)**	40.07 ± 22.94	55.94 ± 21.35	−5.70	<0.01
Self-care	5.34 ± 2.37	6.43 ± 1.85	−3.98	<0.01
Personal/household work	9.71 ± 7.02	14.88 ± 6.57	−5.77	<0.01
Entertainment/social	4.83 ± 3.44	7.13 ± 3.60	−5.28	<0.01
Independent exercise	5.00 ± 4.58	8.14 ± 5.62	−5.85	<0.01
Hand use	11.93 ± 7.12	17.13 ± 6.65	−5.70	<0.01
Leg effort	15.14 ± 10.26	22.92 ± 10.77	−5.96	<0.01
Back effort	4.36 ± 3.15	6.38 ± 2.97	−5.41	<0.01
Wheelchair effectiveness	12.21 ± 6.77	16.62 ± 6.15	−5.30	<0.01

(MAS: maximal activity score, AAS: adjusted activity score)

(*P*-values were obtained from Mann‑Whitney *U* test for continuous variables.)

#### 3.2.3 Comparison of sleep quality differences between patients with diabetes and non-diabetes undergoing maintenance hemodialysis

Data did not conform to normal distribution, so Mann–Whitney *U* tests were used to compare the differences of sleep quality between patients with diabetes and non-diabetes undergoing maintenance hemodialysis. In sleep quality differences, there were significant differences in the scores of each dimension and total scores between two groups. Compared with the patients with non-diabetes, patients with diabetes have higher score of sleep quality (*P* < 0.05) (Table 3).

**Table 3:** Comparison of sleep quality differences between patients with diabetes and non-diabetes undergoing maintenance hemodialysis (Xi'an, China; from April–May 2022).

Characteristic	Patients with diabetes (n = 113)	Patients with non-diabetes (n = 173)	*Z*	*P*
Subjective sleep quality	1.30 ± 0.74	1.10 ± 0.78	−2.20	0.03
Sleep latency	1.55 ± 0.98	1.29 ± 1.00	−2.12	0.03
Sleep duration	1.20 ± 1.01	0.97 ± 0.99	−1.97	0.05
Habitual sleep efficiency	1.21 ± 1.20	0.93 ± 1.13	−1.97	0.05
Sleep disturbances	1.22 ± 0.60	1.02 ± 0.60	−2.45	0.01
Use of sleep medication	0.69 ± 1.10	0.39 ± 0.90	−3.03	<0.01
Daytime dysfunction	1.86 ± 1.06	1.46 ± 1.06	−2.94	<0.01
PSQI total score	9.04 ± 4.48	7.15 ± 4.47	−3.54	<0.01

(PSQI: Pittsburgh Sleep Quality Index)

(*P*-values were obtained from Mann‑Whitney *U* test for continuous variables.)

#### 3.2.4 Comparison of depressive symptoms differences between patients with diabetes and non-diabetes undergoing maintenance hemodialysis

Data did not conform to normal distribution, so Mann–Whitney *U* tests were used to compare the differences of depressive symptoms between patients with diabetes and non-diabetes undergoing maintenance hemodialysis. For depressive symptoms, there was significant difference in the Beck score between two groups. Compared with patients with non-diabetes, patients with diabetes had higher scores of depressive symptoms (*P* < 0.05) (Table 4).

**Table 4:** Comparison of depressive symptoms between patients with diabetes and non-diabetes undergoing maintenance hemodialysis (Xi'an, China; from April–May 2022).

Characteristic	Patients with Diabetes (n = 113)	Patients with Non-diabetes (n = 173)	*Z*	*P*
Beck score	17.32 ± 10.75	12.95 ± 9.26	−3.52	<0.01

(*P*-values were obtained from Mann‑Whitney *U* test for continuous variables.)

### 3.3 Binary multivariate logistic regression

Logistic regression analyses were performed using the Wald test. The presence of MHD duration (OR = 0.99, 95% CI: 0.98, 1.00), AAS (OR = 0.98, 95% CI: 0.97, 0.99), Beck score (OR = 1.04, 95% CI: 1.01, 1.07), and age (OR = 1.02, 95% CI: 1.01, 1.04) were major factors. The Odds Ratio (OR) values of Beck score and age were more than 1. So, depressive symptoms and age were adverse factors for patients with diabetes. Conversely, the OR value of MHD duration and AAS was less than 1, so shorter dialysis duration and higher physical activity (AAS) were protective factors for patients with diabetes undergoing maintenance hemodialysis (Table 5).

**Table 5:** Multiple factors logistic regression on patients with diabetes and non-diabetes undergoing maintenance hemodialysis (Xi'an, China; from April–May 2022).

	*OR*	95% CI
MHD duration	0.99	0.98, 1.00
AAS	0.98	0.97, 0.99
Beck score	1.04	1.01, 1.07
Age	1.02	1.01, 1.04
Constant	0.42	

(MHD: maintenance hemodialysis; AAS: adjusted activity score; OR: Odds Ratio; CI: Confidence Interval)

## 4 Discussion

In our study, 39.51% of patients had diabetes. This proportion is consistent with Tayebeh Soleymanian et al.'s study (41%) (Soleymanian et al., 2017). These findings indicated that diabetic nephropathy is a common cause of ESRD. It has been confirmed that diabetes can aggravate the decline of renal function related to chronic kidney disease (Shanmukham et al., 2022). It is therefore critical to enhance personalized nursing and health intervention programs for this high-risk dialysis population with diabetes. However, given the cross-sectional nature of this study, the observed associations should not be interpreted as causal. The findings indicate statistical relationships rather than temporal or causal effects, and reverse causality cannot be excluded.

In Table 1, compared with patients with non-diabetes undergoing maintenance hemodialysis, patients with diabetes were older and higher proportion of retired patients. And in logistic regression of Table 5, age was an adverse factor for patients with diabetes. Fadwa Al-Ali et al.'s study (Al-Ali et al., 2021) also showed that patients with diabetes undergoing maintenance hemodialysis were older. Advanced age is closely linked to a higher prevalence of chronic comorbidities. Compared with younger populations, elderly individuals often experience declines in physical function and cognitive ability, making them more susceptible to concurrent complications such as diabetes.

In physical activity differences of Table 2, this study showed that compared with patients with non-diabetes; diabetic patients showed a significant reduction of physical activity in patients with diabetes. Aging, long-term decrease in activity, diabetes and chronic kidney disease all can lead to muscle atrophy. Systemic inflammatory reaction, acidosis, use of corticosteroids, neuropathy caused by uremia, hyperparathyroidism, vitamin D deficiency, malnutrition and long-term maintenance hemodialysis treatment can all cause the decline of muscle quality and function of patients with diabetes undergoing maintenance hemodialysis (Jamshidpour et al., 2020). Therefore, effective measures must be taken to improve the physical activity and muscle function of dialysis patients with diabetes. Boshra Jamshidpou et al.'s study showed that increasing the 6-min walking test distance can improve patients' physical function (Jamshidpour et al., 2020).

Our study showed that the patients with diabetes undergoing maintenance hemodialysis have poor sleep quality (Table 3). Our study's results are similar to Fadwa Al-Ali et al.'s study (Al-Ali et al., 2021). Their research showed that dialysis patients with diabetes had significantly more prevalence of poor sleep than those without diabetes (Al-Ali et al., 2021). These results implicated sleep disorders have been reported to be common in patients with diabetes. Poor sleep quality in patients with diabetes can activate the hypothalamus-pituitary-adrenal axis, stimulate the sympathetic nervous system, increase platelet aggregation and inflammatory response, reduce insulin sensitivity, and thus reduce the control and regulation of blood sugar (Dong et al., 2020). Furthermore, impaired sleep quality may stimulate the central nervous system to secrete excessive catecholamines. These hormones can enter the bloodstream and subsequently elevate blood glucose levels (Dong et al., 2020). Conversely, inadequate glycemic control serves as a critical contributor to disrupted sleep quality. Both microvascular and macrovascular complications associated with diabetes—including diabetic retinopathy and cardiovascular disorders—are closely correlated with abnormal sleep duration (Jee et al., 2017).

In depressive symptoms differences of Table 4, compared with patients with non-diabetes undergoing maintenance hemodialysis, patients with diabetes had higher score of depressive symptoms. And in logistic regression of Table 5, depressive symptoms was an adverse factor for patients with diabetes undergoing maintenance hemodialysis. Fadwa Al-Ali et al.'s study also showed that one of the risk factors associated with depression in patients on dialysis is the presence of diabetes (Al-Ali et al., 2021). For patients undergoing maintenance hemodialysis, on the one hand, they need to limit and change their lifestyle such as diet, drinking water, exercise, and so on, and on the other hand, they also have to endure the pain caused by long-term dialysis treatment, which greatly affects the quality of life of patients and can easily lead to the occurrence of depression (Hanspal et al., 2021). In addition, supports from family and the emotional attitudes of patient caregivers also have a significant impact on the psychological well-being of patients (Hanspal et al., 2021). The interconnections between physical activity, sleep quality, and psychological functioning have been previously documented in other populations. For instance, Rezaie et al. reported that better sleep quality and higher physical activity levels predicted lower emotion dysregulation among persons with major depressive disorder (Rezaie et al., 2023). Our findings extend this evidence to the hemodialysis population, suggesting that similar behavioral-psychological interactions may operate in patients with chronic disease, although the cross-sectional design precludes causal inferences.

This study has some limitations. Firstly, the surveys on physical activity, depressive symptoms and sleep quality of patients were all based on self-reports from patients, which may have bias. Secondly, this study was a cross-sectional study and cannot establish causal or temporal relationships. Third, although validated and reliable scales were adopted to assess physical activity, sleep quality and depressive symptoms in this study, these questionnaire-based evaluations cannot replace formal clinical diagnosis. Future research should incorporate objective clinical indicators to further explore the relevant conditions among maintenance hemodialysis patients with diabetes. Fourth, the participants were recruited from a single city, which limits the generalizability of the findings.

## 5 Conclusion

In summary, the present study demonstrated that maintenance hemodialysis patients with diabetes exhibited poorer physical activity levels, impaired sleep quality, and more severe depressive symptoms compared with their non-diabetic counterparts.

It is recommended that hemodialysis centers establish standardized multidisciplinary healthcare workflows to comprehensively improve physical function, sleep status and mental health, and ultimately promote long-term quality of life among this high-risk population.

## CRediT authorship contribution statement

**Lu Zhang:** Writing – review & editing, Writing – original draft, Visualization, Validation, Supervision, Software, Resources, Project administration, Methodology, Investigation, Funding acquisition, Formal analysis, Data curation, Conceptualization. **Sumei Zhang:** Writing – review & editing, Validation, Data curation.

## Ethics declaration

Written informed consent to take part in the study and to publish the article has been obtained from all participants or their legal representatives. The privacy rights of participants have been observed.

This study was performed in compliance with relevant laws, regulatory frameworks and guidelines where the research took place. This study was approved by the Medical Ethics Review Committee of Xi'an Medical University (Approval No. XYLS2024220).

## Declaration of competing interest

The authors declare that they have no known competing financial interests or personal relationships that could have appeared to influence the work reported in this paper.

## Data Availability

Data will be made available on request.
